# Follow-up study regarding the medium-term effectiveness of the home-visiting program “Pro Kind” at age 7 years: study protocol for a randomized controlled trial

**DOI:** 10.1186/s13063-018-2707-3

**Published:** 2018-06-20

**Authors:** Sören Kliem, Malte Sandner, Anna Lohmann, Susan Sierau, Verena Dähne, Annette M. Klein, Tanja Jungmann

**Affiliations:** 1 Criminological Research Institute of Lower Saxony, Lützerodestr 9, 30161 Hannover, Germany; 2 Institute for Employment Research (IAB) of the German Federal Employment Agency (BA), Regensburger Strasse 104, 90478 Nuremberg, Germany; 3 Department of Medical Psychology and Medical Sociology, Universitätsklinikum Leipzig, Philipp-Rosenthal-Str. 55, 04103 Leipzig, Germany; 4 Department of Child and Adolescent Psychiatry, Psychotherapy, and Psychosomatics, Cr, Liebigstraße 20a, 04103 Leipzig, Germany; 5 Institut für Sonderpädagogische Entwicklungsförderung und Rehabilitation (ISER), August-Bebel-Str. 28, 18055 Rostock, Germany

**Keywords:** Home-visiting program, Parenting, Follow-up

## Abstract

**Background:**

Pro Kind is a German adaptation of the US Nurse Family Partnership program. It is an intervention based on home visits targeting first-time mothers from disadvantaged populations. Pro Kind was implemented as a randomized control trial from 2006 to 2012 with *N* = 755 first-time mothers (TG *n* = 394, CG *n* = 391). The 7–8-year follow-up aims to assess the mid-term effects of the program.

**Methods/design:**

Mid-term outcomes are being assessed by trained assessors. In a multimethod approach telephone interviews, on-site interviews, observations and developmental tests will be held in order to assess children’s and mothers’ life satisfaction, mental health, cognitive and social development, parenting behavior, signs of child abuse or neglect as well as the family’s socio-economic status. Furthermore, administrative data will be accessed to obtain information regarding the mother’s usage of pediatric health care, welfare usage and employment history.

**Discussion:**

Results regarding the mid-term effects of the intervention from the Pro Kind Follow-up will provide a scientific basis for future primary prevention programs as well as help stakeholders legitimizing early childhood investments.

**Trial registration:**

German Clinical Trial Registration DRKS-ID, ID: DRKS00007554. Registered on 11 June 2015, updated on 6 October 2017.

**Electronic supplementary material:**

The online version of this article (10.1186/s13063-018-2707-3) contains supplementary material, which is available to authorized users.

## Background

Based on international research findings, home-visiting programs appear to be a promising approach to support psychosocially and/or economically disadvantaged families. Several meta-analyses show that early prevention programs improve parenting skills, reduce child maltreatment and neglect, and have a positive effect on the child’s development [1–8]. Although home-visiting programs have a long tradition in Europe [9], the predominant scientific findings stem from the United States (US), especially from the *Nurse Family Partnership Program* (NFP; [10]). Within the NFP framework, disadvantaged mothers receive close support already before the birth of their first child. The home visits continue up to the child’s second birthday. NFP supports maternal parenting skills relating to the positive support of the child’s emotional, cognitive and social development. Furthermore, child neglect, child maltreatment and child abuse are prevented. By reducing the consumption of alcohol and nicotine, NFP promotes the mother’s health-related behaviors during pregnancy. In addition, the program encourages mothers (or the parents) to expand their formal and informal networks. Finally, the program aims to enable the families to gain financial autonomy and hence independence from unemployment benefits and welfare. To evaluate the NFP program three randomized controlled trials (RCTs) were conducted in 1977, 1988 and 1994 in the three different US populations in Elmira, NY; Memphis, TN; and Denver, CO. In numerous articles these RCTs reported positive effects regarding the mothers’ health-related behavior during pregnancy, the child’s health, parental skills, the socio-emotional and cognitive development of the child, as well as the integration of the mother into the job market or her willingness to take up educational offers. In the long term, better social integration, lower crime rates and improved labor market outcomes were found for children from families who participated in the NFP program [11–30]. According to current estimates (see [31]), approximately 500 child deaths, 4700 abortions, 36,000 cases of partner violence, 90,000 violent crimes by juvenile offenders, 594,000 cases of property crimes or public order disturbances (e.g., vandalism, damage to property), 36,000 prison terms and 41,000 cases of youth substance abuse will have been prevented by NFP in the US by the year 2031. Two independent US research groups conducted comprehensive cost-benefit analyses of NFP. The first analysis, by the Rand Corporation, calculated net returns of US$2.88 for every dollar invested – the returns were greater for the highest-risk families, for which they nearly doubled [32]. The second analysis, by the Washington State Institute for Public Policy, estimated a return of over US$17,000 for every family [33]. Both evaluations factored in the cost savings across several public sectors over 10–15 years, including reduced health care, income assistance and child protection spending. Notably, averted public expenditures were greatest for the most disadvantaged mothers and children, which highlights the importance of offering NFP to those at higher risk [34]. In conclusion, the public savings by NFP in the US (as of 2010) are estimated to be approximately US$1.5 billion [31].

Trials of NFP have also been conducted outside the US (currently, there are also trial registrations for Canada [35] and France [36]). In the Netherlands, a trial that compared NFP to existing health and social services found that it reduced prenatal smoking, increased breastfeeding, reduced the number of child protection reports as well as the incidence of partner violence [37–39]. In contrast, an RCT in England that compared NFP (called FNP) to usual health and social services did not find any additional benefits for either the children or their mothers [40]. These differences in the findings of US, Dutch, and English trials emphasize the necessity of carrying out RCTs in further countries outside of the US before implementing NFP on a larger scale. Results from RCTs would assess the program’s effectiveness as compared to services that are already in place for the target population [41].

### NFP in Germany: The “Pro Kind” study

In Germany, within the framework of the model project Pro Kind, a German adaptation of the NFP program has been scientifically evaluated since 2006. The core components of the NFP interventions were implemented by the Pro Kind program without changes, including the target group criteria, the specified average number of visits and the average duration of a visit. Furthermore, the structured procedure as well as the support for the home visitors through supervision accord with the US original. Modeled after the NFP program, the home visitors conducted the visits regularly, from pregnancy to the child’s second birthday. Diverging from the original program, in the German adaptation home visits were carried out by social workers and state licensed midwives either alone (mainly midwives) or in tandems of a midwife and a social worker (see [42]), whereas in the NFP program exclusively nurses were engaged as home visitors.

### Domains addressed in the Pro Kind intervention

Six focus areas (domains) are addressed while working with the participants in the intervention setting. These domains are generally regarded as the most important risk and protective factors for the prevention of negative pregnancy outcomes, child abuse or neglect, developmental retardation and limited economic independence.

1. Personal health of the mother. Central in this domain is support during pregnancy. Health-related behavior of the expecting mother is addressed such as nutrition, exercise, sleep and rest, as well as oral hygiene. Furthermore, tobacco and alcohol consumption are covered within this domain. Additionally, pregnancy-related physical changes as well as childbirth are discussed.
2. Healthy environment. In this domain the setup of the home environment for parent and child is addressed. Focus aspects are child safety and accident prevention. Toxic or hazardous aspects of the home environment, such as mold and indoor smoking, are also covered.
3. Personal plans for the future. The timing and other considerations of returning to the job or education are the focus of this domain. In this context family planning also plays an important role. Individual preferences, as well as personal strength and weaknesses of the mother, are explored and taken into account for the development of realistic perspectives. Additionally, aspects of everyday personal management, for example, regarding time and money are addressed within this domain.
4. Maternal/ paternal/ parental role. This is the focal domain between birth and the child’s second birthday. But even during pregnancy, parents are sensitized to the basic needs of a child as well as child development in utero. Fears and expectations regarding the life with a child are also addressed. Post-partum baby care and nutrition as well as basic needs of a newly born are covered. Later developmental and educational aspects (i.e., media exposure), as well as stimulation of the parent-child interaction, are important features of this domain.
5. Social network. Social support from the partner, the parents or friends are aspects in this domain. Building interfamilial relationships and friendships as well as falling back on those for everyday support are supported. On the other hand, resolving conflicts, and appropriate non-violent communication between partners are covered.
6. Utilization of social as well as health services. Aspects from this domain are usually covered in case of acute necessity as well as for establishing a general network of support. Examples are check-ups during pregnancy, check-ups for the child or mother-child playgroups or classes. Additionally, mothers are supported in the arrangement or accompanied to official appointments. In cases of psychiatric illness, developmental problems of the child or domestic abuse contact with the relevant social, medical or legal services is facilitated.

### Methodological starting point of the Pro Kind project

#### Sample and sampling

The Pro Kind program was conducted from 2006 to 2012 in the three German states Bremen, Lower Saxony and Saxony (funded by the German Federal Ministry for Family Affairs, Senior Citizens, Woman and Youth BMFSJ [funding code: IIA6-25080820 V6], the Günter-Reimann-Dubbers Foundation [no funding code available], the Dürr Foundation [no funding code available], and the TUI Foundation [no funding code available]; the principal investigator of the intervention phase was TJ). The program was evaluated via longitudinal assessment [43] up to the children’s third birthday within the framework of a multicenter RCT. At the time of the baseline survey, *N* = 755 first-time mothers (TG *n* = 394, CG *n* = 391) participated, who were 12 to 28 weeks pregnant. In addition to first-time pregnancy the inclusion criteria were a financial risk factor (e.g., receiving welfare benefits or being in debt), as well as at least one further social or personal risk factor (e.g., being under age, lack of a school leaving certificate, having experienced abuse or neglect). In an a priori power analysis before the original RCT the sample size was estimated to be *N* = 775. We assumed (1) a minimum detectable effect sizes of ES = 0.20 (i.e., small effect), (2) a type I error rate (false positive) of α = .05, (3) statistical power (1 − *β*) of .80 (where *β* is the probability of making a Type II error, i.e., failing to detect a true effect if it is present) and (4) an attrition rate of 25% for this estimation.

The participants were recruited through various multipliers – especially through gynecologists, midwives, youth welfare offices, psychosocial counseling centers, and employment agencies [44]. After admission to the model project, those who at the time were between 12 and 28 weeks pregnant were randomly allocated to either the study group or the control group via a computer program (Efron’s biased coin design, strata: implementation location, being under age, and nationality of the mothers). Both groups had access to the regular support offered by the German welfare system and were informed about the latter. Furthermore, travel expenses to medical check-ups during as well as after pregnancy were covered as part of the panel maintenance. In addition, compensation was paid for the time participants invested for the program assessment.

#### Program participation

As in the original NFP program, Pro Kind participation was voluntary. It is, hence, evident that not all mothers in the experimental group stayed in the program up to the child’s second birthday and hence not all program contents could be covered as intended. In all, *n* = 166 (42.2%) of the *n* = 394 randomized mothers in the experimental group dropped out of the intervention prematurely. The reasons for termination can be divided into endogenous and exogenous causes. Endogenous termination causes can be understood as those that were caused deliberately and directly by the mother (e.g., through an explicit demand to terminate the program) or indirectly (e.g., by cutting off contact). Conversely, exogenous termination causes can be understood as those caused by external circumstances (e.g., the child being taken into custody by the youth welfare office, sudden death of the child, relocation of the mother to an area where no further home visits could be provided) and hence not deliberately caused by the mother. During the first intervention phase (before the birth of the child) *n* = 52 mothers terminated the program prematurely; hereof, *n* = 38 (73.1%) terminations can be regarded as endogenous terminations. During the second intervention phase (after birth up to the child’s first birthday) *n* = 87 additional mothers dropped out of the intervention. Of these, *n* = 48 (55.2%) can be regarded as endogenous terminations. During the last intervention phase (from the child’s first to second birthday) only 27 more mothers did not complete the program as intended; *n* = 17 (63.0%) due to endogenous termination causes.

Altogether, the families received an average of *M* = 32.7 home visits (*SD* = 18.6) with a range of 0 to 94 home visits. If only those families that completed the program are considered as intended, there was an average of 45.3 home visits (*SD* = 19.7) ranging from 11 to 94 home visits. Across all families, approximately 13,000 home visits were conducted within the Pro Kind project. The average length of a home visit was 82 min. In conclusion, the length of the home visits, as well as the frequency among those families that completed the program, are comparable to those of the US original.

### Current research results from the home-visiting program Pro Kind

Data collection at the time points at 6, 12 and 24 months after birth of the reference child showed small positive effects of the Pro Kind program on maternal feelings of parental self-efficacy (*β* = .03; Wald = 4.05; *df* = 1; *p* = .044), social support (*β* = .04; Wald = 3.85; *df* = 1; *p* = .050) and their knowledge about child-rearing (*β* = .03; Wald = 3.43; *df* = 1; *p* = .064) [43]. In addition, fewer women in the treatment group stated that they suffered from depressive mood 24 months after birth (OR 0.54, *p* = .070]. This finding was confirmed by administrative health insurance data that showed that fewer mothers in the treatment group used antidepressants (*p* = .040^1^). The increased maternal wellbeing may be one reason why in the treatment group fewer pregnancy terminations (conditional on a further pregnancy) than in the control group were observed (OR 0.54, *p* = .090). This might also as a consequence have led to a higher rate of second births 36 months after birth (OR, 1.48, *p* = .030). Regarding the children, the intervention resulted in an improvement in the cognitive development of the girls in the treatment group (at 6 months, ES 0.29, *p* < 0.05; at 12 months, ES 0.28, *p* < .05; at 24 months, ES 0.24, *p* < .01), whereas no improvement could be found in the boys’ cognitive skills. One possible explanation for the gender-specific effect could be that the intended treatment, consisting of the parents reading to, and singing with, their children, was implemented more often with female children compared to male children [45–47].

In addition, the model project Pro Kind is being evaluated by encompassing economic research, for which the most important goal was, and is, to conduct a cost-benefit analysis. The costs for providing the home-visiting program were thereby contrasted with the monetized benefits of the intervention, whereby, in conclusion, the program’s return on investment (ROI) can be determined. The cost of an average intervention was approximately €8.700 [48]. Since the home-visiting program Pro Kind seeks to achieve improvements in several different domains, the saving effects should be different in the various domains and concern different fiscal areas.

## Methods/design

The Follow-Up Study Regarding the Medium-Term Effectiveness of the Home-Visiting Program “Pro Kind,” Based on a Randomized, Controlled Research Design (official study title: “Follow-Up Untersuchung zur mittelfristigen Wirksamkeit des Hausbesuchsprogramms Pro Kind anhand eines randomisierten kontrollierten Forschungsdesigns, Acronym: Pro Kind Follow-up”) is funded by the German Federal Ministry of Education and Research (BMBF funding codes: 01EL1408A, 01EL1408B, 01EL1408C), So far, it has been possible to reestablish contact with a number of families (as of today, approximately 500 families could be reached, re-recruitment is still ongoing, first family contacted on 15 June 2015) and request participation in a follow-up survey. When the (reference) children are at primary school age (7–9 years), the medium-term effects of the home-visiting program Pro Kind is to be studied with the help of multimethod survey approaches from the perspective of the children as well as the mothers, in addition to administrative data. The study is registered in the German Clinical Trial Register (trial ID: DRKS00007554, date of registration: 11 June 2015). The main sponsor of the Pro Kind Follow-up is the Criminological Research Institute of Lower Saxony (KFN, principal investigator Dr. Sören Kliem). Secondary sponsorship is provided by the University of Rostock (Prof. Dr. Tanja Jungmann) the Institute for Employment Research Nuremberg (IAB, Dr. Malte Sandner) as well as the University Clinic Leipzig (Prof. Kai von Klitzing).

### Procedure

**Fig. 1:**
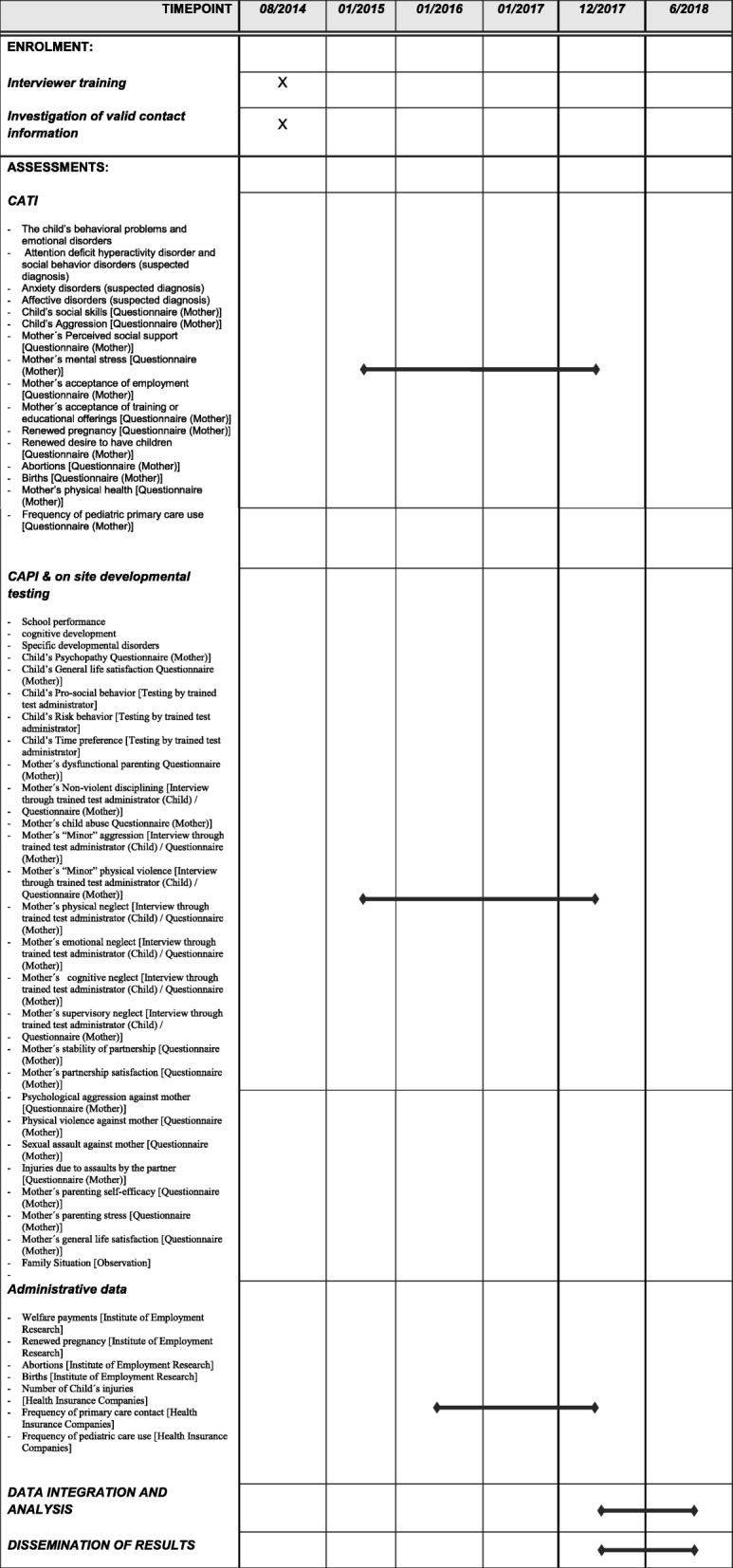
Schedule of Pro Kind Follow-up

The follow-up study can be subdivided in to six domains (1) interviewer training, (2) assessment of contact details, (3) telephone interviews, (4) on-site interviews and developmental tests, (5) acquisition of administrative data and (6) data analysis. The sponsoring institutions will be involved in these domains as follows: KFN: (1), (2), (3), (4), (5); IAB: (1), (2), (5), (6); University of Leipzig: (1), (4), (6), University of Rostock: (1). An overview of the ProKind Follow-up schedule can be found in Fig. 1. The protocol is reported according to the Standard Protocol Items: Recommendations for Interventional Trials (SPIRIT; Fig. 1 and Additional file 1).

#### Interviewer training

All interviewers underwent several days of intensive training as preparation for the home interviews. They received general information regarding child development and background knowledge on the demographic characteristics of the participants. Special focuses of the training were interview and communication techniques including de-escalation methods. Interviewers were sensitized for signs of suicidality and child abuse and instructed how to handle these. They were prepared for problematic situations such as disruptive behavior of the child. The interviewers were certified for the correct application of the measures and tests. Certification was, whenever necessary, provided by external institutions (i.e., for the Kinder-DIPS). All project staff members, including research assistants and interns, were sworn to confidentiality (according to §5 BDSG) upon start of their employment. Should signs for child abuse be uncovered during the study, a conflict between psychological /scientific confidentiality and the obligation to protect the child’s wellbeing might arise. In such a situation, the specific case and the suspicions of the interviewer will be first discussed with the project leader and the necessity to involve third parties evaluated. Before legal action is pursued it will be examined whether personal communication and sensitizing parents regarding the issue at hand as well as encouraging help-seeking behavior in the affected families are sufficient and justifiable (§4 Absatz 1 KKG). It is, furthermore, possible to request anonymous legal advice from local authorities (§4 Absatz 2 KKG). Based on German law, personal information will only be relayed to third parties when the child’s wellbeing is clearly at stake and any attempts of arranging for help remained unsuccessful (§4 Absatz 3 KKG). All members of staff are extensively trained regarding legal matters as well as personal behavior in such situations.

#### Assessment of contact details

Participants are first contacted by the KFN via mail. In this letter participants are informed about the study and can send back a postcard with a current telephone number. In case this initial mail cannot be delivered, the local registration office (Einwohnermeldeamt) is contacted and asked for the current contact details (name and address) of participants. A project assistant then establishes first personal contact via telephone. During this initial phone call appointments for the home visits as well as for the telephone interviews are scheduled.

#### Computer-aided telephone interviews (CATI)

As during the first program phase, scales from the German Socio-Economic Panel (SOEP) [49] as well as the study regarding the health of children and youths in Germany (KiGGS [50]) are used to gather socio-demographic data as well as data regarding the use of health services. In addition, psychometric scales are presented to assess relevant areas that include the mothers’ current living conditions (e.g., the mothers’ general health and social support), as well as that of the children (e.g., general development and socio-emotional development). Furthermore, parts of a fully standardized diagnostic interview, corresponding to the Expert System for Diagnosis of Mental Disorders (Diagnostisches Expertensystem für Psychische Störungen, DIA-X; [51]), are conducted to identify possible mental disorders and problems on the part of the mothers. A trained project team member conducts two telephone interviews with each participant.

#### On-site interviews

In a 2.5-h home visit, two professionally trained project team members conduct structured interviews with the mother and developmental tests with the reference child. These outcome assessors are unaware whether the families are part of the intervention or control group. After an introductory and instruction phase on the part of a project team member, the mother’s consent for testing the children is obtained. All relevant materials are presented to her and she is, furthermore, informed in detail about the test procedures. Subsequently, the *Diagnostic Interview for Mental Disorders for Children and Adolescents* (Kinder-DIPS; [52]) is conducted, whereupon the mother is given a paper-pencil questionnaire to complete. In case an interview or psychological testing is too exhausting for mother or child it is possible to schedule additional appointments to continue the interview and/or testing.

At the same time – with the mother’s consent – the second project team member carries out a structured interview with the child. The children are thereby interviewed regarding their experiences of neglect, based on picture card-based, age-appropriate, structured interviews (Multidimensional Neglectful Behavior Scale, MNBS [53]) and regarding minor physical abuse (Conflict Tactic Scale, CTS-CV [54]). In order to prevent severe stress for the children, only a few, low-intrusive scales are used. No questions are asked concerning experiences of severe violence. The children, furthermore, complete the test battery BUEGA (*Basisdiagnostik Umschriebener Entwicklungsstörungen im Grundschulalter* (Basic diagnostics of specific developmental disorders in elementary school children [55]) and three structured game situations regarding (1) risk behavior according to Dohmen and colleagues [56], (2) pro-social behavior according to Fehr and colleagues [57] and (3) time preference according to Mischel and colleagues [58]. The testing and games that are carried out with the children are entirely age appropriate and playful. Nevertheless, the mere length of the session requires a significant amount of focus and motivation on behalf of the child. It is, hence, possible to divide the tests between at least two sessions should the child show signs of exhaustion or lack of motivation. The test administrator will, together with the mother, make sure that the child’s needs are met and a good relationship of child and test administrator can be established to ensure optimal testing conditions. The children’s participation is entirely voluntary. Children are informed that there will be no negative consequences should they decide not to participate. Furthermore, children are assured that the answers that they provide are confidential. Children are also informed of their right to quit the interview or testing at any moment.

#### Expert judgment

After the on-site interviews, both project team members individually gather observations on site in the domestic environment regarding the living situation, the wellbeing of the child, as well as the quality of the parent-child relationship. Observations are rated according to the scales of the Home Observation for Measurement of the Environment (HOME [59]).

#### Compensation of participants

All participants receive €80 as compensation for the time invested in the telephone interview, face-to face interview and developmental testing. In case only one interview can be conducted the participant receives €30 (telephone interview) or €50 (home visit), respectively. Every child receives a little gift after the home visit (worth approx. €10).

#### Communication of disconcerting test results

The results of the developmental tests (intelligence test, vocabulary test) and diagnostic interviews (DIA-X, Kinder-DIPS) are shared with the parents and explained using an appropriate, yet easily understandable, language. The project staff is available should participants have further questions regarding test results. Upon request project staff members will also assist in the arrangement of counseling or support services for mother or child.

#### Administrative data

In addition to the survey methods mentioned above, administrative data from various sources are used. According to the first project phase, data regarding inpatient and outpatient treatment, as well as medication, aids, mother-child therapies and speech therapy are made available by AOK Niedersachsen, Bremen and Sachsen (German public health insurance company). Here, among other information, it is documented whether vaccinations, early screenings, etc. have been attended at health facilities. Furthermore, information is gathered regarding previous employment, phases of unemployment, and participation in measures during unemployment as well as employment details from the Technical Data Center (FDZ) of the Institute for Employment Research (IAB) of the Federal Employment Agency in Nuremberg. It was possible for the participants to provide self-report data but not grant the researchers access to administrative data.

#### Data handling and monitoring

Data collection and management is overseen by the KFN data manager. He is employed directly by the KFN and not involved in the project either monetarily or scientifically. All project team members have received extensive professional training regarding the application of the measures, data confidentiality but also regarding possible signs of suicidality, child abuse or neglect. All telephone interviews are recorded and evaluated by a second research assistant regarding proper conduct as well as signs of suicidality, child abuse or neglect. Audio- and video-recordings are saved with the primary sponsor and only shared with secondary sponsors for data analysis as stated in the consent form (see Additional file 2). Neither audio nor video transcripts contain personal information beyond the child’s first name and the mother’s initials. After data analysis, video material has to be blurred for alienation purposes or deleted. All outcome assessors were blinded regarding intervention vs. control group membership of the families. Questionnaires were transported in a sealed envelope without identifying personal information beyond a code for longitudinal matching. Personal information was only used for contacting the participants. The personal information is not stored together with the outcome data and is only accessible on a need to know basis.

### Evaluation of effectiveness

**Fig. 2:**
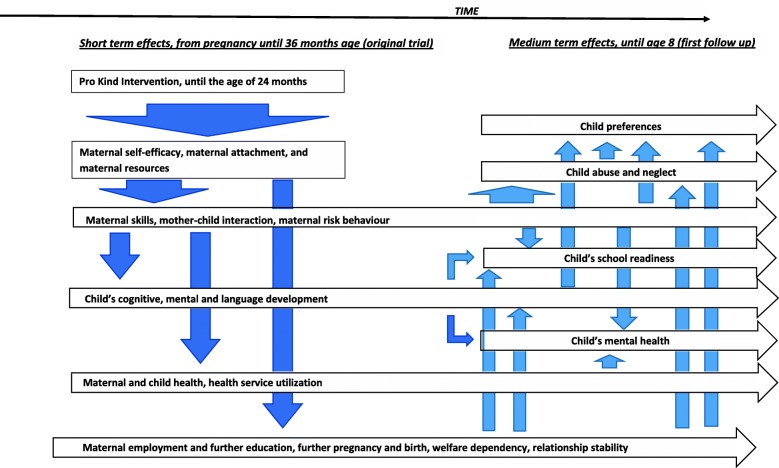
Intervention’s logic model following Olds [10]

The main focus of the scientific work objective is to answer the following research questions (please see Fig. 2 for the intervention’s internal logic. The model shows its hypothesized, causal mechanisms based on a model presented by Olds [10]):

*Research question 1*: *Does the home-visiting program Pro Kind exert a positive effect on the cognitive and socio-emotional development, as well as on the physical health of the child in the medium term?*

- *Hypothesis 1.1*: The home-visiting program has a positive effect on the child’s cognitive development and school performance.
- *Hypothesis 1.2*: The home visits have a positive effect on the child’s mental health.
- *Hypothesis 1.3*: The home visits have a positive effect on the child’s socio-emotional development.
- *Hypothesis 1.4*: The home visits have a positive effect on the child’s life satisfaction.
- *Hypothesis 1.5*: The home visits influence the child’s preferences (risk behavior, pro-sociality and time preference).

*Research question 2*: *Does the home-visiting program Pro Kind, in the medium term, influence the frequency and intensity of parental violence and child neglect?*

- *Hypothesis 2.1*: The home visits result in improved parenting skills (less inappropriate parenting behavior).
- *Hypothesis 2.2*: The home visits reduce or prevent child abuse as well as the frequency of physical violence.
- *Hypothesis 2.3*: The home visits reduce or prevent child neglect.

Research question 3: *Does the home-visiting program Pro Kind have a positive effect on the psychosocial situation as well as on the mothers’ mental health?*

- *Hypothesis 3.1*: The home visits have a positive effect on the mother’s perceived social support.
- *Hypothesis 3.2*: The home visits influence the mother’s mental health.
- *Hypothesis 3.3*: The home visits result in more stable partnerships with less frequent partner change, greater satisfaction with the partnership and less domestic violence in the partnership.
- *Hypothesis 3.4*: The home visits improve the parental self-efficacy expectations concerning the parenting tasks.
- *Hypothesis 3.5*: The home visits have a positive effect on the perceived stress resulting from the mother’s parenting tasks.
- *Hypothesis 3.6*: The home visits have a positive effect on the mother’s life satisfaction.

*Research question 4*: *Does the home-visiting program Pro Kind positively influence the socio-economic situation in the family?*

- *Hypothesis 4.1*: The home visits increase the share of mothers in employment or education programs.
- *Hypothesis 4.2*: The home visits reduce the families’ use of welfare payments (*SGB II, SGB III* and *SGB VIII (social security codes)*).
- *Hypothesis 4.3*: The home visits have a positive effect on the family’s living situation.
- *Hypothesis 4.4*: The home visits influence the timing or frequency of a renewed pregnancy and births.

*Research question 5*: *Does the home-visiting program Pro Kind have a positive effect on the family’s physical health and health service utilization?*

- *Hypothesis 5.1*: The home visits improve the mothers’ physical health.
- *Hypothesis 5.2*: The home visits increase the frequency of pediatric primary care use (e.g., screenings, vaccinations, child’s oral health care and dentist visits).
- *Hypothesis 5.3*: Home visits reduce the children’s hospital visits (outpatient or inpatient) caused by accidents and injuries.

Table 1 briefly describes the procedures used to evaluate the program. It is of special importance whether the underlying constructs are (1) based on a direct efficiency hypothesis of the Pro Kind program (primary outcome) or (2) based on an efficiency hypothesis, but one that must be regarded as less probable than a primary outcome (i.e., secondary outcome) for the following two reasons. First, the intervention did not directly focus on the secondary outcomes, (e.g., child preferences); second, we have less power for the outcomes, (e.g., administrative health services utilization data is only available for one third of the sample as we only cooperate with the largest health insurance company in Germany). Lastly, (3) a construct will be assessed for future research questions but is not regarded as an original aim of the intervention (i.e., no outcome). Due to the amount of data that is to be gathered, in the following only the primary and secondary outcome domains will be presented. The documentation of the study can furthermore be viewed at the German Register of Clinical Trials (DRKS; ID: DRKS000007554. Registration date 11 June 2015; updated on 24 August 2017). In the following, the procedures presented in Table 1 will be described in more detail.

**Table 1:** List of procedures of biopsychosocial evaluation of the Pro Kind Follow-up

Hypothesis	Construct	Operationalization	Primary or secondary outcome domains	Informant	Data source
*Hypothesis 1.1*: The home-visiting program has a positive effect on the child’s cognitive development and school performance	Child’s school performance	Basic diagnostics of specific developmental disorders of speech and language at primary school age (BUEGA [55])	Primary outcome domain	Child	Testing by trained test administrator
	Child’s cognitive development				
	Child’s specific developmental disorders				
*Hypothesis 1.2*: The home visits have a positive effect on the child’s mental health	Child’s behavioral problems and emotional disorders	Child Behavior Checklist (CBCL 6/18 R [60]), German version: [61]	Primary outcome domain	Mother	Questionnaire
	Child’s attention deficit hyperactivity disorder and social behavior disorders (suspected diagnosis)	Module from the diagnostic interview of mental disorders in children and youths (Kinder-DIPS [52])		Mother	Interview by trained and certified staff
	Child’s anxiety disorders (suspected diagnosis)				
	Child’s affective disorders (suspected diagnosis)				
*Hypothesis 1.3*: The home visits have a positive effect on the child’s socio-emotional development	Child’s social skills	Social Skills Improvement System (SSIS [62]), German version: author’s translation	Secondary outcome domain	Mother	Questionnaire
	Child’s aggression	Questionnaire regarding children’s aggressive behavior (FAVK [63])		Mother	Questionnaire
	Child’s psychopathy	Inventory of Callous-Unemotional Traits (ICU) ([64]), German version by Essau		Mother	Questionnaire
*Hypothesis 1.4*: The home visits have a positive effect on the child’s life satisfaction.	Child’s general life satisfaction	Inventory to measure the life quality of children and youths (ILK [65])	Primary outcome domain	Child	Survey by trained test administrator
*Hypothesis 1.5*: The home visits influence the child’s preferences (risk behavior, pro-social behavior and time preference).	Child’s pro-social behavior	Game for interpersonal allocation decisions ([57]).	Secondary outcome domain	Child	Testing by trained test administrator
	Child’s risk behavior	Investment decisions in a lottery ([56])			
	Child’s time preference	Game for temporary allocation decisions ([58])			
*Hypothesis 2.1*: The home visits result in improved parenting skills (less inappropriate parenting behavior)	Mother’s dysfunctional parenting	Parenting Scale (PS) ([66]), German version: target-group-specific adaptation by the authors	Primary outcome domain	Mother	Questionnaire
	Mother’s non-violent disciplining	Conflict Tactic Scale Child Report (CTS-CR [68]), interview of the children with picture cards. German version: translation by the AMIS group / Conflict Tactic Scale Parent Child (CTS-PC [67]), German version: target-group-specific adaptation by the authors		Child / Mother	Interview through trained test administrator (Child) / Questionnaire (Mother)]
*Hypothesis 2.2*: The home visits reduce or prevent child abuse and the frequency of physical violence	Mother’s child abuse	Conflict Tactic Scale Parent Child (CTS-PC) ([54]), German version: target-group-specific adaptation by the authors	Primary outcome domain	Mother	Questionnaire
	Mother’s “Minor” aggression	Conflict Tactic Scale Child Report (CTS-CR [68]), interview of the children with picture cards. German version: translation by the AMIS group / Conflict Tactic Scale Parent Child (CTS-PC) ([54]), German version: target-group-specific adaptation by the authors		Child / mother	Interview through trained test administrator (Child) / Questionnaire (Mother)
	Mother’s “Minor” physical violence				
*Hypothesis 2.3*: The home visits reduce or prevent child neglect	Mother’s physical neglect	Scale of the Multidimensional Neglectful Behavior Scale-Child Report (MNBS [53]), interview of the children with picture cards. German version: translation by the AMIS group.	Primary outcome domain	Mother	Questionnaire
	Mother’s emotional neglect			Child / mother	Interview through trained test administrator (Child) / Questionnaire (Mother)
	Mother’s cognitive neglect				
	Mother’s supervisory neglect				
*Hypothesis 3.1*: The home visits have a positive effect on the mother’s perceived social support	Mother’s Perceived social support	Questionnaire regarding social support (FSOZU-K6 [71])	Secondary outcome domain	Mother	Questionnaire
*Hypothesis 3.2*: The home visits influence the mother’s mental health	Mother’s mental stress	Depression-Anxiety-Stress Scale (DASS [70]), German version: target-group-specific adaptation by the authors	Primary outcome domain	Mother	Questionnaire
*Hypothesis 3.3*: The home visits result in more stable partnerships with less frequent partner change, greater satisfaction with the partnership and less domestic violence in the partnership	Mother’s stability of partnership	Developed by the authors	Secondary outcome domain	Mother	Questionnaire
	Mother’s partnership satisfaction	Short form of the Partnership Questionnaire (PFB-K [74])			
	Psychological aggression against mother	Conflict Tactics Scales (CTS2 [54]; German version: target-group-specific adaptation by the authors [forward-backward]			
	Physical violence against mother				
	Sexual assault against mother				
	Injuries due to assaults by the partner				
*Hypothesis 3.4*: The home visits improve the parental self-efficacy expectations regarding the parenting tasks	Mother’s parenting self-efficacy	Parenting Sense of Competence Scale (PSOC) ([76]), German version: target-group-specific adaptation by the authors	Secondary outcome domain	Mother	Questionnaire
*Hypothesis 3.5*: The home visits have a positive effect on the perceived stress resulting from the mother’s parenting tasks	Mother’s parenting stress	Parenting Stress Index (PSI [72]), German version: Eltern-Belastungs-Inventar (EBI [73]).	Secondary outcome domain	Mother	Questionnaire
*Hypothesis 3.6*:The home visits have a positive effect on the mother‘s life satisfaction	Mother’s general life satisfaction	Questions regarding life satisfaction (FLZ [77])	Primary outcome domain	Mother	Questionnaire
*Hypothesis 4.1*: The home visits increase the share of mothers in employment or education programs	Mother’s acceptance of employment	The German Socio-Economic Panel (SOEP) [49] and	Secondary outcome domain	Mother	Questionnaire
	Mother’s acceptance of training or educational offers	The Panel Arbeitsmarkt und Soziale Sicherung ([78])		Mother	Questionnaire
Hypothesis 4.2: The home visits reduce the families’ use of welfare payments (SGB II, SGB III and SGB VIII [social security codes]).	Welfare payments	Integrated employment history provided by the Institute of Employment Research (IAB)	Secondary outcome domain	Institute of Employment Research (IAB)	Administrative data
Hypothesis 4.3: The home visits have a positive effect on the family’s living situation	Family situation	The Home Observation for Measurement of the Environment (HOME [59] forward-backward translation by the authors)	Secondary outcome domain	Staff	Observation
Hypothesis 4.4: The home visits influence the timing or frequency of a renewed pregnancy and births	Renewed pregnancy	Questionnaire about intended and realized fertility (Siedler et al., 2009). Integrated Employment History provided by the Institute of Employment Research (IAB)	Secondary outcome domain	Mother/ Institute of Employment Research (IAB)	Questionnaire/ administrative data
	Renewed desire to have children				
	Abortions Births				
Hypothesis 5.1: The home visits improve the mothers’ physical health	Mother’s physical health	12-Item Short Form Survey (SF-12 [79])	Secondary outcome domain	Mother	Questionnaire
Hypothesis 5.2: The home visits increase the frequency of pediatric primary care use (e.g., screenings, vaccinations, child’s oral health care and dentist visits)	Frequency of pediatric primary care use	KiGGS questionnaire [80] physician visits with ICD Z	Secondary outcome domain	Mother health insurance companies	Questionnaire/administrative data
Hypothesis 5.3: Home visits reduce the children’s hospital visits (outpatient or inpatient) caused by accidents and injuries	Number of child’s injuries	Hospital admission and physician visits with ICD S and T	Secondary outcome domain	Health insurance companies	Administrative data

### Deployed measuring instruments

#### Research question 1: The child’s development

##### School performance and specific learning disability

The Basic Diagnostics of Specific Developmental Disorders in Elementary School Age Children (BUEGA; [55]) is an elementary school test battery that aims to measure relevant specific developmental and attention disorders in elementary school children (6–10 years old). It is also possible to use the procedure to evaluate the school achievement potential. Furthermore, an aptitude profile can be created that illustrates the child’s strengths and weaknesses. The BUEGA consists of seven subtests: *Verbal intelligence* (52 items), *Non-verbal intelligence* (38 items), *Expressive language* (57 items), *Reading* (32 items), *Spelling* (10 to 18 items, depending on the grade level), *Arithmetic* (40 items) and *Attentiveness* (strike-through test). The reliabilities for the different scales are between Cronbach’s α = .79 and .96.

##### Mental health

The Child Behavior Checklist (CBCL 6/18 [60]) measures behavioral problems, emotional problems, somatic complaints, as well as social competences of school-age children and youths from their parent’s perspective. The eight syndrome scales (*withdrawn/depressed*, *somatic complaints*, *anxious/depressed*, *social problems*, *thought problems*, *attention problems*, *rule-breaking behavior* and *aggressive behavior*) can be summed up to scales of internalizing and externalizing disorders as well as to an overall sum score. Appropriate internal consistencies of Cronbach’s α > .80 are reported in the German manual [61] for the *total problem score* and the scales *internalizing behavior* and *externalizing behavior.*

When dealing with mental disorders in children and adolescents, the Diagnostic Interview for Children and Youths (Kinder-DIPS; [52]) allows for a diagnosis of mental disorders according to DSM-IV and ICD-10. It encompasses both a parental and a children’s version (for children from the age of 6 years). It allows the assessment of a wide spectrum of current and lifetime mental disorders in children and adolescents: *attention problems*, *activity problems* and *social problems*, *tic disorders*, *anxiety disorders*, *elimination disorders*, *sleep disorders*, *affective disorders* and *eating disorders.* Furthermore, it contains a general clinical-demographic part as well as several screenings (e.g., for *alcoholism*, *drug abuse,* as well as *non-organic psychosis)*. In addition, a psychiatric history, a family history of mental disorders as well as Axes IV (*Psychosocial and environmental problems*) and V (*Global measurement of the level of functioning*) can be assessed. To measure the occurrence of symptoms, the frequency of occurrence or the intensity of a symptom is coded on a 4-point rating scale (*never/seldom* to *very often*, or *never* to *very strong*). For the individual disorder categories, the interrater reliabilities of the parental version (long-term diagnoses) lie between Kappa = .48 (*sleep disorders*) and Kappa = .88 (*affective disorders*). The following modules of the parental version are to be covered within the present study: attention-deficit hyperactivity disorder, anxiety disorders and affective disorders.

#### Socio-emotional development

##### Social competence

The Social Skills Improvement System (SSIS [62]; in a translation by the authors adapted to the target group (forward-backward) makes it possible to measure the social skills of children and youths between the ages of 3 and 18 years. The SSIS instrument consists of several scales to measure three domains: *Social Skills* (subscales*: Communication*, *Cooperation*, *Assertion*, *Empathy*, *Engagement* and *Self-Control*), *Problem Behaviors* (subscales: *Externalizing*, *Internalizing*, *Hyperactivity/Inattentiveness*, *Autism Spectrum* and *Bullying*) and *Academic Competence* (subscales: *Reading*, *Mathematics*, *Motivation*, *Support by parents* and *General Cognitive Function*). In the present study, the domain *Social Skills* will be included as part of the maternal assessment.

##### Aggressiveness

The German questionnaire for aggressive behavior in children (FAVK, “Fragebogen zum aggressiven Verhalten von Kindern” [63]) assesses triggering and maintaining components of aggressive behavior. The external assessment version for parents, teachers and educators will be filled out from the mother. A distinction is made between aggressive behaviors and cognitions toward peers and adults. The FAVK consists of 25 items with a 4-point rating scale on how applicable the behavior is (ranging from *not applicable at all* to *particularly applicable*). Four facets of aggressive behavior are measured: *social-cognitive information processing disorders*, *impulse control disorders*, *social skills disorders* and *social interaction disorders*. The internal consistency of the questionnaire ranges between Cronbach’s α = .92 and .95.

##### Psychopathy

The Inventory of Callous-Unemotional Traits (ICU) [64] measures callous, cunning and hard-hearted qualities and is based on the *Callous Unemotional* scale of the APSD. The ICU consists of three scales: *Callousness, Uncaring* and *Unemotional* and contains a total of 24 items. The teachers are to assess the occurrence of behaviors by children on a four-stage scale (*not at all true* to *definitely true*). The documented internal consistency of the ICU is between Cronbach’s α = .64 and .77.

##### Life satisfaction

To measure the life quality of children and youths a German inventory is administered (“Inventar zur Erfassung der Lebensqualität bei Kindern und Jugendlichen”, ILK [65]). Life quality is divided into different areas that are measured separately: *school*, *family*, *social contact with peers* and *interests and recreational activities.* Furthermore, there are two health-related topics: *physical health* and *mental health*. In addition to the individual areas, an overall assessment of quality of life is possible. The entire self-assessment version of the ILK (apart from the health-oriented areas) is to be used when interviewing the child. Seven items are thus presented with an answer format suitable for children (Smiley symbols). The internal consistency of the life quality scores for children and youths range between Cronbach’s α = .55 and .63.

#### Children’s preferences

##### Pro-social behavior

The game regarding interpersonal allocation decisions [57] consists of four rounds. In each round, the child can choose between an egalitarian allocation and an alternative where the child gains an advantage or disadvantage over other imaginary children. In the *pro-social decision situation*, the child can choose between the allocation (1–1), which means 1 point for the child and 1 point for the imaginary other children, and the allocation (1–0). This allocation allows the child to distribute a point to the other imaginary children without additional costs. In the *cost-incurring pro-social decision situation*, the child is to choose between a (1–1) and (2–0) allocation. The child can only increase their own payment at the expense of the imaginary children. In the *envy decision situation*, the child chooses between the allocation (1–1) and (1–2), which makes it possible for the child to reduce the payment to the imaginary children without incurring any costs of their own. In the *cost-incurring envy decision situation* the child chooses between the allocation (1–1) and (2–4), which leads to a reduction of the payment to the imaginary children that incurs costs of their own.

##### Risk behavior

The game *Investment decision in a lottery* [56] measures the child’s willingness to take risks. Before each round the child can bet between zero and five points in a lottery, in which they can double their points with a probability of 50% or lose the points they have bet with a probability of 50%. Points that are not bet are safe for the child. The number of points that are bet can be regarded as a measure of the child’s willingness to take on risk.

##### Time preference

The game regarding *temporary allocation decisions* [58] makes it possible to assess the child’s individual time preference. In this game, the child can choose whether they want to immediately receive the points won in the previous game, exchange them for a present or continue to collect further points by deciding to receive the present at a later point in time. The child receives 2 points for every week that they waits. The child can wait for a maximum of 4 weeks. The number of weeks can be regarded as a measure of the individual time preference.

### Research question 2: Dysfunctional parenting behavior, child abuse and child neglect

#### Dysfunctional parenting behavior

The Parenting Scale (PS [66] in a translation by the authors adapted to the target group (forward-backward)) measures dysfunctional disciplinary parenting behavior. Based on examples of children’s behavior, the parents are to classify their own behavior ranging between *functional* and *dysfunctional*.

#### Child abuse

The Parent-Child Conflict Tactic Scales (CTS-PC [67] in a translation by the authors adapted to the target group (forward-backward)), is a parent-child version of the CTS that measures psychological and physical abuse and neglect, as well as non-violent disciplining of children by their parents. The instrument contains 35 items and can be used when interviewing the mother, whereby she rates her own behavior toward her child. The CTS-PC is composed of the subscales *nonviolent discipline*, *psychological aggression*, *physical assault*, *neglect*, *weekly discipline* and *sexual abuse*.

The CTS-PC, Child Report assesses conflict resolution strategies in family or close relationships as rated by the children. In line with the parent-version, the child version measures psychological and physical abuse and neglect, as well as nonviolent disciplining of children by their parents. It is designed for 6- to 9-year-olds. It operates with pictures that describe conflict situations in the family that the child may possibly recognize. The pictures depict different levels of severity, whereby only the three dimensions non-violent discipline, psychological aggression and minor physical assault are used in the present study. A psychometric evaluation of the adapted German version of the CTS-PC was recently conducted by the AMIS (analyzing pathways from childhood maltreatment to internalizing symptoms and disorders in children and adolescents) study of the University of Leipzig [68].

#### Child neglect

The Multidimensional Neglectful Behavior Scale (MNBS [69]; German version translated for the AMIS study of the University of Leipzig) assesses various forms of parental behavior regarding child neglect. There are various versions of the MNBS, and all of them measure the extent to which the following needs of a child have been neglected: *Physical needs* (e.g., food and clothing), *emotional needs* (e.g., affection and support), *supervisory needs* (e.g., taking an interest in the child’s misconduct and knowledge about their whereabouts) and *cognitive needs* (e.g., reading out loud or helping with homework). The MNBS exists, among other versions, as a self-report for parents and children aged 0–15 years (Form P/PS). This version is to be used when interviewing the mother. With its 38 items, it measures her neglectful behavior when raising her child. Furthermore, within the present study, a picture-based children’s version is presented. The picture cards are thereby shown to the child, who chooses situations that are applicable to their everyday family life. The children are presented with the three scales *emotional neglect, cognitive neglect* and *supervisory neglect*.

### Research question 3: Maternal mental health

#### Stress

The short form of the Depression-Anxiety-Stress Scale (DASS-21 [70]; a translation adapted to the target group (forward-backward)) serves to assess the frequency (*never* to *very often*) of negative emotional states during the last 4 weeks. The DASS-21 contains three dimensions: *depression* (e.g., dysphoria and hopelessness, *anxiety* (e.g., autonomic arousal and situational anxiety) and *stress* (e.g., chronic, non-specific arousal and irritability).

#### Psychopathology

The DIA-X [51] is a fully structured diagnostic procedure to measure mental disorders based on ICD-10 and DSM-IV criteria. The interview is a modified form of the CIDI (Composite International Diagnostic Interview) from the World Health Organization (WHO). The DIA-X makes it possible to survey more than 100 mental disorder categories. It gathers data on *organic mental disorders*, *substance disorders*, *affective disorders (manias, hypomanias, depressive disorders)*, *anxiety disorders*, *obsessive-compulsive disorders*, *reactions*, *dissociative disorders*, *somatoform disorders* and *eating disorders.* Lifetime as well as acute diagnoses can be obtained. The DIA-X is divided into 16 sections. Due to the possibility of selecting and presenting individual sections of the interview, an adapted interview that focuses on specific disorders can be carried out. The instrument comprises four DIA-X screening questionnaires: one stem screening questionnaire, which contains all 16 stem questions in the DIA-X interview, an anxiety screening questionnaire, a depression screening questionnaire, and a questionnaire regarding premenstrual syndrome. These questionnaires make it possible to assess whether it is any indication to conduct the entire DIA-X interview. The interrater reliabilities are specified as being between the values of Kappa = .81 to 1.0.

#### Social support

The short form of the questionnaire regarding perceived social support (FSozU-K 6 [71]) measures the social support as perceived or anticipated support from the social environment. The items are in the form of statements, whereby the participant specifies their level of agreement on a 5-point scale (*not at all true* to *absolutely true)*. The internal consistency of FSozU-K6 was found to be Cronbach’s α = .90.

#### Stress due to parenting tasks

The Parenting Stress Index (PSI) [72] (German version: Eltern-Belastungs-Inventar, EBI [73]) assesses whether parents, due to an increased level of stress, are impaired regarding their care and support of their child. The EBI contains a total of 48 items that are answered on a 5-point Likert scale (*absolutely true* to *not true at all)*. The internal consistency of the EBI overall scale amounts to Cronbach’s α = .95.

### Partnership

#### Partnership satisfaction

The short form of the partnership questionnaire (PFB-K; [74, 75]) assesses the satisfaction in a partnership or marriage with the help of nine items. Three items can be allocated to each of the three subscales *fighting behavior, tenderness* and *commonality/communication*. Within a representative population survey, an internal consistency of Cronbach’s α = .84 (women: Cronbach’s α = .87; men: Cronbach’s α = .81) was determined for the overall value of the PFB-K.

#### Partnership violence

The Conflict Tactics Scales (CTS2) [54] translation adapted to the target group (forward-backward)) serves to assess conflict resolution strategies within the family or close relationships. The CTS2 (revised and modified version of the CTS) measure three forms of conflict handling: *negotiation* (six items), *psychological aggression* (eight items) and *physical violence* (12 items). In addition, it measures *sexual coercion* (seven items) and *injuries caused by violence by the partner* (six items). With the help of CTS2, information is collected about one’s own behavior as well as that of the partner. The latter is relevant to the present study.

#### Partnership stability

The items assessing partnership stability document the marital status of the participant (unmarried, married, widowed or divorced). Additionally, the participants are asked for household composition, whether she is in a partnership and whether the partner is the biological father of the treatment child.

#### Self-efficacy

The Parenting Sense of Competence Scale (PSOC [76] translation adapted to the target group [forward-backward]) measures the experience of competence in the role as parent on two dimensions: *satisfaction* and *efficacy.* The dimension *satisfaction* comprises nine items and includes *parental care, motivation* and *frustration.* The dimension *efficacy* measures *parental competency, performance* and *problem-solving ability* with the help of seven items. The 16 items are presented as statements and assessed on a six-point Likert scale (*strongly disagree* to *strongly agree*).

#### Life satisfaction

The German Version of the Life Satisfaction Questionnaire (“Fragebogen zur Lebenszufriedenheit”, FLZ [77]) serves to measure relevant aspects of life satisfaction in ten areas of life (*health, work and career, financial situation, recreation, marriage and partnership, relationship with one’s children, own self, sexuality, friends/ acquaintances/ relatives* and *housing*). Each subscale comprises seven items, which are assessed on a 7-point rating scale (*very dissatisfied* to *very satisfied*). The questionnaire can be used from the age of 14 and will be used when interviewing the mother. In addition to measuring the area-specific life satisfaction, the FLZ allows for the assessment of the general life satisfaction, which is calculated as the sum score of seven of the ten scales. The internal consistency for the overall value are satisfactory (mothers α = .74; fathers: α = .76). The FLZ was standardized via a representative German sample, hence norms are available for various age and occupational groups.

### Research question 4: The families’ socio-economic situation

#### Family income, employment and education

Different scales from the German Socio-Economic Panel (SOEP) [49] were used to asses family income, employment rates and educational status. The German SOEP study (GSOEP) is conducted by the *Deutsches Institut für Wirtschaftsforchung* (DIW – German Institute for Economic Research). It was initiated in 1984 and has been conducted annually since then.

#### Acceptance of employment, training or educational offers

A five-item scale used in the SOEP [49] as well as in the *Panel Labor Market and Social Security* (PASS [78]) was used to measure employment attitudes. These questions contain certain statements about the importance of employment for the individual to which the participant can completely agree or completely disagree. Additionally, a six-item scale used in the SOEP and PASS assesses acceptable obstacles regarding the start of an occupation. The respondents can rate these difficulties on a 4-point Likert scale from *I will accept in any* case to *I will not accept in any case*.

#### Welfare payments

The Research Data Centre (FDZ) of the Federal Employment Agency at the Institute of Employment Research (IAB) provides social security data (Integrated Employment History) for all participants. The participating individuals will be identified by the participants’ name, date of birth and address from the social security data. For all participants who are identified via their social security data, it is possible to merge survey and assessment data with their welfare receipt spells, amount of welfare, and additional cash transfers. Additionally, the social security data provides employment spells, wage spells and indicators about the type of occupation. The data is available from the participant’s entry into the labor market until the time of the follow-up data collection.

#### Family situation

The Home Observation for Measurement of the Environment (HOME [59] forward-backward translation by the authors) assesses the lifestyle and living situation within a family. As an external evaluation, following the home-visiting, the interviewer reports whether, based on yes-no answers, certain circumstances (e.g., regarding the apartment) or behaviors (e.g., regarding the interaction with the child) could be observed in the family or not. *Yes* identifies the existence and *No* the non-existence of the circumstance or behaviors specified by each item. Depending on the age of the children, the HOME consists of 45 to 60 items. The subscales also vary depending on the version. The HOME exists in four versions for the assessment of the living situations of children of different age groups. Due to some questionable items (e.g., negative rating for the lack of a television), the items were culturally adapted to the German-speaking area as well as to family life in the 21st century in general.

### Research question 5: Health and health service utilization of mother and child

#### Mother’s physical health

The 12-item Short Form Health Survey (SF-12 [79]) is an outcome-oriented, subjective measure of physical and mental health. It includes 12 items. Ten items are assessed on a 5-point Likert scale asking how often (*always* to *never*) different physical and mental stresses and strains are absent or present in the previous 4 weeks. Additionally, two questions assess whether the health status decreases (*strongly*, *a little bit, not at all*) the ability to climb stairs and lift. Anderson et al. [79] show that the SF-12 is a suitable instrument to conduct health economic quality of life calculations. As the SF-12 is widely used in other panel surveys, it provides the possibility for external validation of the participant’s responses.

#### Child physical health and health service utilization

The German Health Interview and Examination Survey for Children and Adolescents (KiGGS) questionnaire [80] assesses accidents which lead to outpatient or inpatient care in the last 12 months before the survey. In the case of an accident, the location and the type of the accident are inquired of, as well as the injury sustained. For all these questions standardized answers are presented. Furthermore, the KiGGS questionnaire inquires the frequency of certain diseases as well as the utilization of certain health care services. An alternative source for the assessment of pediatric primary care usage (e.g., screenings, vaccinations) is the evaluation of health insurance data. For those participants who declared consent, data can be merged to the participants by the individual health insurance number. With the help of the International Statistical Classification of Diseases and Related Health Problems (ICD) code, it is possible to identify the date and frequency of pediatric primary care utilization.

#### Number of injuries

The health insurance data can also be used to identify injuries that lead to a hospital admission or physician visit. All injuries and intoxications are coded S and T in the ICD systematic. Due to the health insurance data, it is possible to identify the date of the injury, the duration of treatment and which follow-up treatments are needed which can serve as a proxy for the severity of the injury.

### Data analysis

The primary and secondary analyses will be conducted on an intention-to-treat basis. Multilevel modeling will be used for statistical analyses of our primary and secondary outcomes. Prior to analysis, data will be checked for outliers, inconsistencies and possible transformation. We predict that our sample size will be large enough for our statistical tests to be robust regarding non-normally distributed variables. To reduce bias and loss of statistical power in our analysis of primary and secondary outcome indicators, we will use multiple imputations to estimate values for missing data. Power analyses (i.e., *sensitivity*) based on an attrition rate of 30% (*N* = 542 for the CATIs, CAPIs and developmental tests) given a type I error rate (false positive) of α = .05 and statistical power of 1− *β* = .80 resulted in a minimum detectable effect sizes of ES = 0.2. For an attrition rate of 40% (*N* = 465) a minimum detectable effect sizes of ES = 0.23 was estimated. To take that into account we analyze different outcome measures within one hypothesis, we will adjust our results for multiple hypothesis testing (MHT) to control the family-wise type I error rate.

#### Subgroup analysis

We will conduct preplanned analyses for different participants’ and child characteristics contributing to positive effects in the original trial (see [43, 45–47]). For this purpose, we consider the influence of different factors (i.e., maternal age at pre-assessment and number of mother-related risk factors at pre-assessment and child gender). Additionally, we will analyze subsequent births as a mediating factor of treatment efficacy in a preplanned subgroup analysis as a greater number of second births in the treatment group was a main effect in the original trial. Due to earlier findings [45], all primary and secondary outcomes will be analyzed with regard to any differential effects, while controlling for children’s gender.

## Discussion

The aim of the study is to conduct a follow-up survey regarding the effectiveness of the German adaptation of the NFP program, approximately 6 years after the intervention ended. The following variables are to be tested regarding the medium-term effectiveness of the home-visiting program: (1) prevention of mental and physical illnesses in mothers as well as children, (2) strengthening of parenting skills and prevention of child abuse and neglect, (3) improvement of child’s cognitive development and school performance, (4) strengthening child’s socio-emotional development, (5) positively influencing the socio-economic situation of the family, (6) positively influencing the psychosocial situation of the family and (7) reduction of the costs to the health and social system.

NFP’s medium-term effectiveness has, however, not previously been tested in Europe. This poses a problem, not least because NFP constitutes a time-limited intervention that is implemented prenatally as well as over the first 2 years of the child’s life. It should, however, be noted that findings from NFP trials in the US indicate that significant benefits can increase over time and that new developmental advantages may arise as the children grow older. As an example, many of the most conclusive findings regarding NFP have been found as late as 5 to 20 years after the end of the intervention. This includes a reduction in the child’s level of anxiety, depression and substance abuse symptoms; a reduction in severe antisocial behavior during adolescence; and a decrease in both child and maternal mortality from preventable causes [13, 14, 25]. Due to these later occurring effects, a benefit-cost ratio greater than 1 does not emerge before age 5 years [32]. Bearing this in mind, this RCT was designed to allow for long-term follow-up – both through strong retention efforts and by including measures that predict the long-term outcome, such as the cognitive development and behavior of the child. This means that whatever successes or failures of NFP can be identified as a result of the outcome of trials conducted when children turn 2 years old, these findings may be invalidated during the children’s subsequent periods of development.

### Limitations

Selective data loss can lead to a skewed assessment of intervention effects. However, non-selective data loss also has adverse consequences, such as a decrease in the statistical power. In order to ensure maximum participation of the families who were contacted, a double-staged recruiting strategy is pursued: To obtain the missing contact data, in a first step, access is gained to data from the residents’ registration offices and the employment agencies. In a second step, the successfully contacted families are offered financial incentives for a renewed participation in the study. Furthermore, contact with the participants was continuously maintained between project phases. For example, participants regularly received birthday and Christmas cards as part of panel maintenance.

### Implications

Although studies from the US confirm the effectiveness of NFP, these results cannot simply be transferred to European countries. Overall high-quality replications (i.e., RCTs) of the US studies are scarce (with the exception of RTCs in the UK, France and The Netherlands). Especially for Germany, this seems problematic as here comprehensive support of comparable programs is provided with public funding (e.g., the Federal Government supported the “Netzwerk Frühe Hilfen” (National Center for Early Support) from 2012 to 2015 with up to €177 million), even though a comprehensive and long-term assessment of the corresponding programs has yet to be undertaken. In spite of a large number of early childhood interventions and special home-visiting programs with limited methodological quality in Germany [81], no medium-term RCT has been conducted to examine the effectiveness of home-visiting programs within the early childhood support in Germany.

#### Ethical approval

The Ethics Committee of the German Society for Psychology (DGPs; Registration No.: SK 122014), as well as the Ethics Committee of the University of Leipzig authorized the study’s design and procedure (406–14-15,122,014). A data protection concept is available that received positive appraisal by the responsible data protection officer in Lower Saxony (2.2–1759-240). Participation in the interviews and the testing is voluntary. No disadvantages will result if the participant decides not to agree to an interview. Withdrawing from the study is possible at any time, verbally or in writing. If a participant withdraws her consent, all data that identify her are deleted and the remaining data concerning the participant will be processed anonymously. All participants will be informed in an appropriate form and in simple language about the results of the studies that are conducted. The project staff are available to answer any questions. Should there be any unusual findings, the participants will be supported in receiving counseling or other assistance for herself and her child.

#### Dissemination

After completion of data assessment, the anonymous data will be shared among the cooperating institutions. As further follow-ups are planned (up to the children’s 20th birthdays), access to the data (including video- and audio-recordings, that will be blurred for alienation or deleted after evaluation) will remain limited to the scientist from the cooperating institutions. Dissemination of the results to the professional as well as the lay public is pursued by publications in peer-reviewed journals, project reports as well as with the help of the interdisciplinary network “Nationales Zentrum Frühe Hilfen (NZFH – National Center for Early Support)” which is an important platform for early childhood interventions and prevention in Germany. Results from the Pro Kind Follow-up will provide a scientific basis for future primary prevention programs as well as help stakeholders legitimizing early childhood investments.

## Trial status

Collection and assessment of contact addresses began in September 2014. The first mothers have been contacted in October 2014 to assess their willingness to participate in the follow-up. The first telephone interviews have been held in April 2015. Data collection is expected to be completed by 31 December 2017.

## Additional files

Additional file 1: Standard Protocol Items: Recommendations for Interventional Trials (SPIRIT) Checklist. (PDF 169 kb)

Additional file 2: Model consent form. (PDF 503 kb)

## Abbreviations

AMIS: Analyzing pathways from childhood maltreatment to internalizing symptoms and disorders in children and adolescents
BMFSJ: German Federal Ministry for Family Affairs, Senior Citizens, Woman and Youth
BUEGA: Basic diagnostics of specific developmental disorders in elementary school children
CAPI: Computer-assisted personal interview
CATI: Computer-aided telephone interviews
CBCL: Child Behavior Checklist
CTS-CV: Conflict Tactic Scale
CTS-PC: Parent-Child Conflict Tactic Scales
DASS-21: Short form of the Depression-Anxiety-Stress Scale
DGPs: German Society for Psychology
DIW: German Institute for Economic Research
EBI: Eltern-Belastungs-Inventar
FAVK: German questionnaire for aggressive behavior in children
FDZ: Technical Data Center of the IAB
FLZ: German Version of the Life Satisfaction Questionnaire
FSozU-K 6: Short form of the questionnaire regarding social support
HOME: Home Observation for Measurement of the Environment
IAB: Institute for Employment Research
ICU: Inventory of Callous-Unemotional Traits
ILK: Inventar zur Erfassung der Lebensqualität bei Kindern und Jugendlichen
KFN: Criminological Research Institute of Lower Saxony
KiGGS: German Health Interview and Examination Survey for Children and Adolescents
Kinder-DIPS: Diagnostic Interview for Mental Disorders for Children and Adolescents
MNBS: Multidimensional Neglectful Behavior Scale
MNBS: Multidimensional Neglectful Behavior Scale
NFP: Nurse Family Partnership Program
NZFH: National Center for Early Support
PASS: Panel Labor Market and Social Security
PFB-K: Partnership questionnaire
PS: Parenting Scale
PSI: Parenting Stress Index
PSOC: Parenting Sense of Competence Scale
ROI: Return on investment
SF-12: Short Form Health Survey
SOEP: German Socio-Economic Panel

## Footnotes

1 No odds ratio can be calculated because there was no event in the intervention group. The *p*-value is based on the relevant percentage points (linear model).
