# Supplementary material for: Follow-up study regarding the medium-term effectiveness of the home-visiting program “Pro Kind” at age 7 years: study protocol for a randomized controlled trial
Source: Trials. 2018 Jun 20;19:323. doi: 10.1186/s13063-018-2707-3 (PMC6011474; doi:10.1186/s13063-018-2707-3)
Supplement: Supplementary file 2 — Model consent form. (PDF 503 kb) [file 13063_2018_2707_MOESM2_ESM.pdf]

Participant information regarding the study

Welcome to the follow-up survey for the pilot Pro-Kind program. We thank you for your continuing interest in the study.

As during our visits and telephone conversations in the past, we would like to know how you are doing and how your living situations have changes. Furthermore, we are interested in the developmental progress of your child regarding different developmental areas.

Study process

As you already know from our previous assessments we would like to ask you several questions **over the phone**. During the phone conversation we will ask you questions regarding your present living situation (e.g. *Did you have another child? Have you started or finished school or vocational training? What does your social environment look like?)* Furthermore, we would like to know more about possible burdens in your everyday life (*e.g. stress in the workplace, vocational training, or the family*) and how this might have affected your overall health (e.g. *Are you full of energy or do you just need a break? Are you sick often?)* as well as your emotional wellbeing (e.g. *Are you usually happy or often sad? Do you sleep well or not? Do you feel left alone? Are you looking to the future with confidence?*). Additionally, we would like to ask questions regarding your child's behavior and emotions (*e.g. Is he/she friendly or also aggressive from time to time? Does he/she have many or only few friends? Is he/she often happy or rather sad?).*

The telephone interview will last approximately **2 hours** and will be held during **two sessions (one hour each).** The reimbursement for the telephone interview will be **50€**.

At another time we would like to conduct an **interview with you and your child at your home**. We would like to know how you have experienced raising your child in the past as well as at present. (e.g. *Were there times where you felt overwhelmed and helpless? Were there Times where you could not fulfill your child's needs? Did you seek and receive help? Were there joyous times with your child/children where you experienced motherhood as especially fulfilling? In general, what advice would you like to give other mothers?)*

We would like to carry out some tests with your child (e.g regarding language development, vocabulary and coping with conflicts). We would also like to ask your child how he or she experiences everyday life with you (*e.g. Does he/she always feels treated fairly? Does he/she feel that you are always there for him/her? Does he/she feel loved? What does your child like about you? Is your child happy with his/her school? What is your child interested in?)*. Of course, all questions will be explained to you beforehand and your consent will be obtained. Should your child need a break, we will incorporate that.

Two staff members will visit you at home. The visit will take approximately **2 ½ hours**. Reimbursement for the home visit will be **50€.** Your child can furthermore choose a little present from the list you will find attached. We will bring this **present** with us when we come to your house.

Voluntariness and anonymity

Your participation in the study is of course **voluntary**. You may at any time and without explanation withdraw your consent. Terminating your participation will not result in any negative consequences.

Data and personal information obtained as part of this study are **confidential** and handled as such. All members of staff that have your personal details due to being in touch with you personally have been sworn to secrecy.

In some rare circumstances, it can happen that during the course of the study family problems come to the surface, that might have consequences regarding custody (e.g. suspicion of child abuse or neglect). In this case we would first contact you personally and try finding a solution for you and your child. This might include in some occasions consultations with child-care services.

Protection of data privacy

The data assessment is carried out without any mentioning of your name. Your answers and test scores will be stored under a number (pseudonymisation). With the help of a code list, your name can be linked to your number. This code list is password protected and only members of staff have access to it. The list will be deleted as soon as all the data is collected and no further follow-up is planned. At the present follow-up assessments up to your child's 20th birthday are planned. After the deletion of the code list, the data is completely anonymous and cannot be linked to you anymore in any way. In this anonymized form the data will be stored for at least 10 years. As long as the coding list has not been deleted and the data not been anonymized you can request the deletion of your data at any time.

Video and audio recordings

The testing of your child will be video-recorded. The personal interview as well as the telephone conversations with you will be audio-recorded. These recordings are only used for assessment and quality assurance. The recordings are hence made to ensure and check the conduct of staff members only and by no means monitor your behavior or that of your child. Handling of the recordings is in strict accordance with the data protection act. Video and audio material will be stored without mentioning any names and archived for at least 10 years after the completion of the study. Recordings will only be handed over to third parties in anonymized form and only for the purpose of quality assurance. Third parties will delete the recordings immediately after quality assessment.

Follow-ups

For follow-ups we will contact administrative offices, health insurance providers or the Institute of Employment Research (IAB) in case your contact details are not up to date.

Reimbursement

For your participation in the telephone interview **as well as** in the home visit you will be reimbursed with **100€.** The reimbursement can be paid to you in cash or wired to your account. In case of a cash payment, you will have to sign a receipt including your name and address. In case of a bank transfer, you will need to state your bank details. All information in this regard will be stored separately from any data relating to the study.

General consent form

For the participation in the research project

Follow-Up Study Regarding the Medium-Term Effectiveness of the Home Visiting Program “Pro Kind,” Based on a Randomized Controlled Research Design

I (name in capital letters)

____

Have been instructed, orally as well as in writing, about the nature, meaning, risks and consequences of this this study as well as the assessment. I had enough time for consideration. I have read and understood all information. Should I have had any questions regarding the study, Ms __________________ has answered them completely and to my satisfaction.

I consent to the described handling of the data that has been obtained during the study. The recordings as well as the data assessment will be carried out pseudomized i.e. by using a number and not mentioning my name. I am aware of the existence of a coding list that can link my name to this number. The coding list is password protected and only assessable by project staff. It will be deleted after the completion of data assessment. I am aware that I can withdraw my consent regarding data storage without any disadvantage. I have been informed that can I can request the deletion of any personal and identifying data at any time. I approve of the storage of the completely anonymized data as well as the usage of the same for research purposes for at least 10 years. I have been informed that apart from legal obligations no personal or identifying data may be handed over to a third party. I know that the present study is carried out in cooperation with other researchers. I consent to the recording of the data relating to this study in the form of questionnaires and electronic media, as well as the dissemination in pseudomized form to the following parties:

a. The Criminological Research Institute of Lower Saxony for scientific assessment
b. Cooperating researchers of the University of Rostock, the Department of Child and Adolescent Psychiatry, Psychotherapy and Psychosomatics, University of Leipzig, as well as the Institute of Employment Research (IAB);
c. The responsible authorities (Federal Ministry of Education and Research, Berlin) for the inspection of the proper conduct of the study.

Consent form for the participation of my child

I hereby permit that my child may take part in the assessment during which age appropriate interviews, tests and games will be conducted. All interviews and tests have been presented and explained to me. Visual material has been shown to me. I will not be shown the answers my child provided unless they were related to results that indicate abnormalities that possibly require treatment (see also below).

I consent to the above

O YES O NO.

Audio and Video recordings

I permit video recordings to be made of my child during testing. Furthermore, the assessment of myself may be audio recorded. These recordings are for quality assurance as well as for the support of the quantitative testing.

I am aware that these recordings will be saved and archived without the mentioning of names. They may be used for analysis, quality assurance and supervision purposes only. The project staff has been sworn to secrecy and are bound by data protection laws. I know that the recordings as well as other assessment results will be archived for at least 10 years after completion of the study to ensure research integrity and transparency. I am aware that the recordings will be evaluated by independent experts. Those experts only evaluate the conduct of the staff members and not my own or my child's behavior. The recordings will only be passed on to third parties in anonymized form. After the assessment of the interviewer conduct, third parties will immediately delete the recordings.

I consent to the above

O YES O NO.

Contacting teachers for assessment

My child's teachers may be sent a set of questionnaires. These questionnaires contain questions regarding my child's behavior in school. The teacher will not receive any information regarding the study and the study purpose.

Name of teacher:_______________________________

Name of school:____

I consent to the above: O YES O NO.

Consent for obtaining administrative Data

I approve, that as during the first phase of the project- health care providers as well as the association of statutory health insurance physicians may be contacted to obtain information regarding health care utilization for me or my child. I furthermore agree that information regarding diagnoses and other health issues may be obtained from health care providers as well as the association of statutory health insurance physicians.

I consent to the above: O YES O NO.

I furthermore permit, that data may be obtained from the Institute of Employment Research (IAB) of the German Federal Employment agency. This includes for example information regarding employment or phases of unemployment. For obtaining this information, my contact information may be sent to the IAB. The IAB will delete my contact data after linking the required information. I furthermore allow the IAB to pass on my present contact details to the KFN for follow-up studies.

I consent to the above: O YES O NO.

I furthermore permit that results of the school entry examinations may be obtained from the local health care administration.

I consent to the above: O YES O NO.

Feedback regarding results that may indicate abnormalities that warrant treatment.

Should any psychiatric abnormalities reading yourself or psychiatric abnormalities, learning disabilities and attention deficits of your child become apparent during the assessment, we will inform you. You are free to consult further experts regarding these suspicions and obtain professional help. Should you request that, we can provide you with further information.

Feedback regarding Child development

Testing of my child will provide vast insights regarding his/her development. After the assessment I would like to receive written information regarding my child's psychological development.

O YES O NO

Continuation of the study.

I give consent, that in case of a follow-up study my personal details might be used to reestablish contact. I have been informed that this data will be stored until the final termination of the study in a coding list and that only project staff members have access to it. After 20 years at most my personal data will be deleted. Until then I am entitled to request information regarding my personal data and demand their deletion.

I consent to the above: O YES O NO.

I had enough time for consideration and am willing to participate in the above-mentioned study. I know that participation is voluntary and that I can terminate at any given time without providing any justification. I know that in case of termination I will still be entitled to reimbursement for the participation up to that point.

I have received a copy of this consent form including participant information, which are considered part of the consent form.

Place, Date & Participant's signature: ____________________

Name of participant in block letters: ____________________

Place, Date & signature of other legal guardian ____________________

Name of other legal guardian in block letters: ____________________

In case no other legal guardian is present the participant assures, that the other legal guardian agrees to the testing of the child.

Place, Date & signature of participant ____________________

Place, Date & Signature of the investigator ____________________

Name of investigator in block letters: ____________________

Study information and consent form for the child.

The child will be informed in a child appropriate conversation conveying the following content:

Hallo, my name is ______________________.
It is nice to meet you and I am looking forward to spending some time with you today.
Maybe your mom has already told you that we are going to ask you a few questions and play with you a little bit.
We are researchers, do you know what a researcher is? *(wait for child's reaction, then continue)*

Some researchers try to find out how big the earth is or how high the world's highest mountain is. Some would like to know which is the smallest particle or why people get sunburned.
We would like to know what children need so they can develop well. "Develop well" means that they get along in school, get along with their friends and parents and are happy and satisfied most of the time.
To find out more about that we ask mothers, teachers but also children a lot of questions. So, your opinion matters a great deal to us. It is just as important as, for example, your mother's opinion.
In a few minutes I have some questions and some pictures for you. I will always take the time and tell you exactly why I am doing what I am doing and explain to you what you should do.
You can ask me anytime, in case you did not understand something. Okay? *(wait for child's reaction, then continue)*

Would you like to participate? *(wait for child's reaction, then continue)*
If I ask you a question that you would rather not answer, that is fine. You don't have to. You won't be punished for that and no one will be angry with you. And of course, you can still keep your present. However, I would of course love for you to answer all the question to the end. We won't tell anybody what you answered. We will also not tell you what your mother told us. But you can tell your mom if you want to it does not have to be a secret.
Please, tell me right away if you don't like something or don't want to do something. Please, also tell me if you would like to take a break, don't want to answer a question or would rather stop altogether.

If you are okay with me asking you some questions now, please put your name here.

________________________________ (child's name)
